# Mechanism of action and therapeutic use of bempedoic acid in atherosclerosis and metabolic syndrome

**DOI:** 10.3389/fcvm.2022.1028355

**Published:** 2022-10-28

**Authors:** Gianni Biolo, Pierandrea Vinci, Alessandro Mangogna, Matteo Landolfo, Paolo Schincariol, Nicola Fiotti, Filippo Mearelli, Filippo Giorgio Di Girolamo

**Affiliations:** ^1^Medical Clinic, Department of Medical, Surgical and Health Sciences, Cattinara Hospital, University of Trieste, Trieste, Italy; ^2^Institute for Maternal and Child Health – IRCCS “Burlo Garofolo”, Trieste, Italy; ^3^Hospital Pharmacy, Cattinara Hospital, Azienda Sanitaria Universitaria Giuliano Isontina, Trieste, Italy

**Keywords:** bempedoic acid, atherosclerosis, cholesterol, statins, ezetimibe, PCSK9 inhibitors, hsCRP, inflammation

## Abstract

Bempedoic acid is a new cholesterol-lowering drug, which has recently received US FDA and EMA approval. This drug targets lipid and glucose metabolism as well as inflammation *via* downregulation of ATP-citrate lyase and upregulation of AMP-activated protein kinase (AMPK). The primary effect is the reduction of cholesterol synthesis in the liver and its administration is generally not associated to unwanted muscle effects. Suppression of hepatic fatty acid synthesis leads to decreased triglycerides and, possibly, improved non-alcoholic fatty liver disease. Bempedoic acid may decrease gluconeogenesis leading to improved insulin sensitivity, glucose metabolism, and metabolic syndrome. The anti-inflammatory action of bempedoic acid is mainly achieved *via* activation of AMPK pathway in the immune cells, leading to decreased plasma levels of C-reactive protein. Effects of bempedoic acid on atherosclerotic cardiovascular disease, type 2 diabetes and chronic liver disease have been assessed in randomized clinical trials but require further confirmation. Safety clinical trials in phase III indicate that bempedoic acid administration is generally well-tolerated in combination with statins, ezetimibe, or proprotein convertase subtilisin/kexin type 9 (PCSK9) inhibitors to achieve low-density lipoprotein cholesterol targets. The aim of this narrative review on bempedoic acid is to explore the underlying mechanisms of action and potential clinical targets, present existing evidence from clinical trials, and describe practical management of patients.

## Introduction

In 2019, the European Societies of Cardiology and Atherosclerosis published a new version of the guidelines (1, 2) to reduce cardiovascular risk by controlling dyslipidemia in patients with atherosclerotic cardiovascular disease (ASCVD) (3) and/or with heterozygous familial hypercholesterolaemia (HeFH) (4). The new guidelines include emerging evidence from several placebo-controlled randomized clinical trials (RCT) on the effects of monoclonal antibodies to proprotein convertase subtilisin/kexin type 9 (PCSK9). PCSK9 inhibitors added to statin therapy further reduce the risk of ASCVD. This effect is directly correlated with the absolute reduction in low-density lipoprotein cholesterol (LDL-C). For patients with very high cardiovascular risk due to manifest ASCVD, the proposed LDL-C target value is <55 mg/dl (1.4 mmol/L), while for patients with high cardiovascular risk it is <70 mg/dl (1.8 mmol/L). To reach such low levels of LDL-C it is necessary to optimize drug therapy using a combination of the available cholesterol-lowering drugs, provided there is not an LDL-C level under which a subject can be considered safe from ASCVD. Statins should be prescribed in high intensity formulations (atorvastatin 40–80 mg or rosuvastatin 20–40 mg), allowing a LDL-C reduction by about 50%, whereas the combination with ezetimibe provides a further reduction of 15% (Table 1). Anti-PCSK9 monotherapy leads to about 60% reduction of LDL-C. The combination of high intensity statin, ezetimibe, and PCSK9 inhibitor is potentially able to reduce LDL-C by 85% compared to baseline values, allowing the achievement of the therapeutic target in most cases (Table 1). In the real world, however, it is clear that more than 80% of patients who require therapy with cholesterol-lowering drugs for either primary or secondary prevention of cardiovascular diseases do not reach the targets set in the 2019 guidelines (5, 6). Failure to achieve those targets is associated to a significant increase in the number of cardiovascular events in the whole population.

**TABLE 1:** Effectiveness of cholesterol-lowering treatments.

Treatment	Average LDL-C reduction (%)
Moderate intensity statin	≈ 30
High intensity statin	≈ 50
High intensity statin plus ezetimibe	≈ 65
PCSK9 inhibitor	≈ 60
PCSK9 inhibitor plus high intensity statin	≈ 75
PCSK9 inhibitor plus high intensity statin plus ezetimibe	≈ 85
Bempedoic acid	≈ 30
Bempedoic acid in maximally tolerated statin	≈ 15
Bempedoic acid plus ezetimibe	≈ 45
Bempedoic acid plus moderate intensity statin plus ezetimibe	≈ 60

Adapted from the 2019 European Societies of Cardiology and European Societies of Atherosclerosis Guidelines for the management of dyslipidemias (1, 2) and from references (26, 27, 29, 30).

Poor adherence to statin therapy is the main reason for failure to achieve therapeutic targets. A recent meta-analysis evaluated the results of 8 RCTs involving 1,766,385 statin users (7). Across all studies, 42% (interquartile range, 37–48%) of patients discontinued the treatment. The discontinuation of statins was mainly due (83% of cases) to the development of myalgia, while an increase in creatine phosphokinase (CPK) plasma levels was observed only in 11% of the cases. In most cases, statin-associated muscle symptoms (SAMS) consist in myalgia without objective signs of muscle inflammation (8). In few cases, muscle inflammation develops with a significant increase in plasma CPK levels. Only in sporadic cases, severe forms of rhabdomyolysis with acute renal failure are observed. The PROSISA study involved 23 Italian lipid clinics and enrolled 16,717 patients treated with statins. The study showed that 9.6% of patients reported SAMS (9). Statin de-challenge (i.e., therapy interruption) and re-challenge (i.e., change and restart of statin treatment) led to recurrence of muscular symptoms only in 38%, suggesting that myalgia is reversible and often not directly caused by statin therapy.

Furthermore, some studies suggest an association among SAMS, elderly and exercise. Thompson et al. evaluated CPK levels in patients taking lovastatin or placebo after a treadmill bout (10). They found that CPK was 62% higher (*p* < 0.05) in the lovastatin group after adjusting for initial CPK differences. In another controlled study, CPK levels of 37 volunteers treated with statins were measured before and after running the 2011 Boston Marathon. Authors found that CPK concentrations were directly related to age in statin users, but not in the controls, suggesting that susceptibility to exercise-induced muscle injury with statins increases with age (11). Indeed, in cases of poor adherence to statin therapy or in special cluster of patients, such as master athletes (12) or when physical therapy is strongly suggested (13, 14), one of the other existing alternative therapies can be considered to reach the lipid-lowering goals.

While discontinuation of statin therapy is very frequent, adherence to PCSK9 inhibitors therapy is high. Nonetheless, subcutaneous self-injection of these drugs is not acceptable for some patients. Furthermore, bureaucratic and prescriptive constraints as well as costs can limit their use. Together with poor therapeutic adherence also underestimation of cardiovascular risk by both healthcare professionals and patients lead to failure in reaching therapeutic targets. Two new cholesterol-lowering drugs, which have recently received US FDA and European EMA approval, could foster therapeutic adherence (15, 16). Inclisiran is an inhibitor of PCSK9 synthesis with a biannual subcutaneous administration performed by the healthcare professional. Bempedoic acid (ETC-1002) is a drug taken orally that significantly reduces all atherogenic lipid markers, including LDL-C, triglycerides and apolipoprotein B. Furthermore, the drug shows a strong anti-inflammatory effect decreasing high sensitivity C-reactive protein (hsCRP) levels. Despite a mechanism of action partly overlapping with that of statins, bempedoic acid administration ordinarily is not associated to unwanted muscle effects.

## Mechanism of action

**FIGURE 1:**
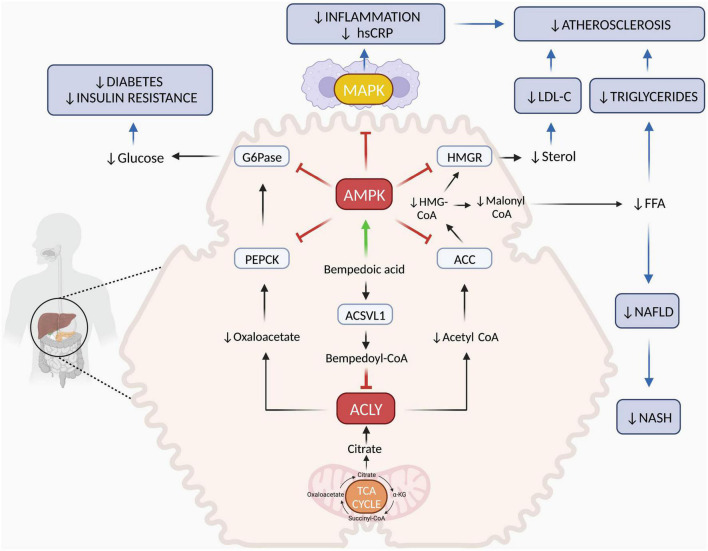
Mechanisms of action of bempedoic acid. In the cytoplasm of hepatocytes, ACSVL1 converts bempedoic acid to bempedoyl-CoA that directly inhibits ACLY. This is a key enzyme responsible for generating acetyl-CoA and oxaloacetate from citric acid deriving from mitochondrial TCA cycle. Reduction of acetyl-CoA and oxaloacetate levels affects fatty acid and cholesterol synthesis as well as gluconeogenesis. In addition, bempedoic free acid activates the AMPK pathway, which downregulates key rate-limiting enzymes of lipid synthesis (ACC and HMGR) and glucose production (PEPCK and G6Pase). In the liver, the combination of ACLY inhibition and AMPK activation by bempedoic acid decreases cholesterol, FFA and glucose synthesis both by reducing precursor substrates and by downregulating key enzyme activities. Through this mechanism, bempedoic acid may improve dyslipidemia, hepatic steatosis, and diabetes. In skeletal muscle, bempedoic acid cannot be activated to bempedoyl-CoA because ACSVL1 is completely absent in this tissue. Thus, this drug does not promote myotoxicity associated with cholesterol synthesis inhibition, as statins can. In the immune cells and in other tissues, AMPK activation by bempedoic acid downregulates MAPK pro-inflammatory pathways leading to decreased cytokine, chemokine, and adhesion molecule synthesis. Through this mechanism, bempedoic acid decreases inflammation, hsCRP levels and may contribute to atherosclerosis and NASH prevention. Red T indicate inhibition; green arrow indicates activation; blue arrows indicate clinical targets. α-KG, α-ketoglutarate; ACLY, ATP-citrate lyase; ACSVL1, very long-chain acyl-CoA synthetase-1; AMPK, AMP-activated protein kinase; ACC, acetyl-CoA carboxylase; HMGCoA, 3-hydroxy-3-methylglutaryl coenzyme A; HMGR, 3-hydroxy-3-methylglutaryl-CoA reductase; G6Pase, glucose-6-phosphatase; PEPCK, phosphoenolpyruvate carboxykinase; TCA, tricarboxylic acid; FFA, free fatty acids; NAFLD, non-alcoholic fatty liver disease; NASH, non-alcoholic steatohepatitis; MAPK, mitogen-activated protein kinase; hsCRP, high-sensitivity C-reactive protein. Figure was created with BioRender.com.

The beneficial effects of bempedoic acid on metabolism and inflammation are closely linked to the modulation of two key enzymes that regulate lipid, carbohydrate and energy metabolism (17). First, bempedoic acid inhibits hepatic ATP-citrate lyase (ACLY), an enzyme that regulates the flow of extra-mitochondrial citrate in the synthesis of lipids. Second, it upregulates the AMP-activated protein kinase (AMPK), a pivotal kinase that controls energy homeostasis and inflammation at the whole organism level. The combination of these two molecular actions results in strong effects on LDL-C synthesis and systemic inflammation with additional benefits on the metabolic syndrome and diabetes. The mechanism of action of bempedoic acid is described in Figure 1.

### Downregulation of ATP-citrate lyase

Bempedoic acid is a pro-drug that is converted in the liver into the active form, bempedoyl-CoA, by the very long-chain acyl-CoA synthetase-1 (ACSVL1), an enzyme present in the hepatocyte but completely absent in skeletal muscle. The action of the active form of bempedoic acid consists in the inhibition of the enzyme ACLY (17–19). This enzyme is at the intersection between pathways of glucose and lipid metabolism, regulating gluconeogenesis and lipogenesis. Bempedoyl-CoA inhibits *de novo* sterol and fatty acid synthesis through the direct inhibition of ACLY. Cytoplasmic ACLY converts the citric acid deriving from mitochondrial Krebs cycle into oxaloacetate and acetyl-CoA. Decreased availability of oxaloacetate reduces gluconeogenesis and hepatic glucose production. Acetyl-CoA is precursor of both lipogenesis and sterol synthesis. Lipogenesis is achieved through the formation of malonyl-CoA by the enzyme acetyl-CoA carboxylase (ACC) and the subsequent synthesis of fatty acids and triglycerides. The synthesis of cholesterol is achieved through the formation of 3-hydroxy 3-methylglutaryl-CoA (HMG-CoA) and mevalonic acid mediated by the enzyme HMG-CoA reductase (HMGR) which is target of the statins. The effect of bempedoic acid on cholesterol synthesis is similar to that of statins but acts at a higher level on the metabolic synthesis pathway. Inhibition of hepatic cytoplasmic ACLY by bempedoyl-CoA causes suppression of cholesterol synthesis and a compensatory increase in membrane receptors for LDL leading to increased LDL clearance from the blood. ACSVL1 is not expressed in skeletal muscle, impeding the conversion of bempedoic acid into its active form. Therefore, in skeletal muscle, bempedoic acid should not promote toxicity associated with cholesterol synthesis inhibition. In Mendelian randomization studies, genetic variants that mimic the effect of ACLY inhibition were associated with reductions in serum biomarkers of cardiovascular risk such as triglycerides and LDL cholesterol, non-alcoholic fatty liver disease (NAFLD), body fat and type 2 diabetes (20, 21). These results are consistent with a relevant inter-individual variability of ACLY expression (22), which might affect efficiency of gluconeogenesis and effectiveness of bempedoic acid. Thus, pharmacogenetics of ACLY inhibitors is a potential scenario of personalized medicine.

### Upregulation of AMP-activated protein kinase

In addition to inhibition of liver ACLY by bempedoyl-CoA, bempedoic acid directly activates AMPK, which is a master regulator of whole-body glucose and energy homeostasis. AMPK activation downregulates key rate-limiting enzymes of gluconeogenesis such as glucose-6-phosphatase (G6Pase) and phosphoenolpyruvate carboxykinase (PEPCK). G6Pase hydrolyzes glucose 6-phosphate resulting in free glucose ready to be exported from the cell, whilst PEPCK catalyzes the first step of gluconeogenesis converting oxaloacetate into phosphoenolpyruvate. In addition, ACLY inhibition contributes to decrease glucose production by reducing oxaloacetate availability for gluconeogenesis. In addition to the effects on glucose metabolism, AMPK inhibits the rate-limiting enzymes of fatty acid and cholesterol synthesis pathways, i.e., ACC and HMGR, respectively. Thus, bempedoic acid targets two distinct pathways in lowering liver fatty acid and cholesterol synthesis. In addition to the metabolic actions, bempedoic acid significantly reduces circulating hsCRP *in vivo* through strong systemic anti-inflammatory effects. In *LDLR*^–/^*^–^* mice, fed with a high-fat diet, bempedoic acid supplementation reduced plasma and tissue lipid elevations, attenuated the expression of proinflammatory genes, suppressed cholesteryl ester accumulation in the aortic wall with the consequence of preventing the development of atherosclerotic plaques (23). *In vitro*, bempedoic acid regulated the inflammatory response of activated monocytes by enhancing the anti-inflammatory AMPK pathway and inhibiting the mitogen-activated protein kinase (MAPK) pro-inflammatory pathway, leading to reduced release of proinflammatory cytokines and chemokines (24). In a mouse model of diet-induced obesity, bempedoic acid restored adipose AMPK activity, decreased adipose tissue inflammation and reduced interleukin-6 synthesis (24). Additionally, inhibition of ACLY by bempedoic acid reduced prostaglandin E2 production and contributed to decrease the inflammatory response (25).

## Clinical targets

### Dyslipidemia

Phase II RCT indicates that bempedoic acid administration at the standard dose of 180 mg/day to patients with hypercholesterolemia but unable to tolerate statins results in reductions of approximately 30% in LDL-C (26, 27). The cholesterol-lowering effect of bempedoic acid tends to be more pronounced in patients with type 2 diabetes (28). The effects of administering bempedoic acid in combination with ezetimibe, a cholesterol intestinal absorption inhibitor, are additive, resulting in a 45% reduction in LDL-C (27). Bempedoic acid and statins act on two different enzymes of the same cholesterol synthesis pathway. Inhibition of ACLY by bempedoic acid reduces precursor availability for HMGR and cholesterol synthesis. Consequently, we do not expect additive effects of a combined therapy with the two drugs. In fact, the administration of bempedoic acid enhances the cholesterol-lowering action of high-intensity statin therapy by only about 15% as compared to statin alone (29). Combination of bempedoic acid 180 mg, ezetimibe 10 mg, and atorvastatin 20 mg in patients with hypercholesterolemia decreased LDL-C by about 60% (30). An additive effect of bempedoic acid on LDL-C reduction was also observed in patients receiving PCSK9 inhibitor therapy. Bempedoic acid (180 mg once daily) added to background therapy with subcutaneous evolocumab (420 mg once monthly) significantly lowered LDL-C by about 30% (31). It is known that the administration of cholesterol synthesis inhibitors such as statins and bempedoic acid causes an increase in the expression of PCSK9, a protein that increases circulating LDL-C levels by increasing the degradation of the LDL-C receptor. When bempedoic acid and statins are used in combination with a PCSK9 inhibitor, the inhibition of PCSK9 enhances the effects of cholesterol synthesis inhibitors on LDL receptor overexpression leading to a synergistic LDL-C lowering action. In addition to decreased LDL-C, the inhibition effect of fatty acid synthesis results in the reduction of plasma levels of free fatty acids and triglycerides. Meta-analysis of phase II and III RCTs confirmed that bempedoic acid significantly reduced triglycerides by 15% and HDL-C by 6% (32).

### Liver steatosis

Non-alcoholic fatty liver disease is presently the most frequent cause of chronic liver injury. NAFLD can develop into steatohepatitis (NASH), a condition which in addition includes inflammation, hepatocellular lesions and fibrosis and can progress to liver cirrhosis and hepatocellular carcinoma. The development of NAFLD is strongly associated with the metabolic syndrome. Both disorders predict type 2 diabetes and cardiovascular diseases. There is no currently approved treatment for NAFLD or NASH. It would be of great clinical relevance to find a drug with positive effects on liver steatosis and, at the same time, on risk factors for type 2 diabetes and cardiovascular diseases. In humans and animal models, elevated hepatic *de novo* lipogenesis contributes to NAFLD (33). ACLY is upstream of ACC regulating cytosolic acetyl-CoA availability. Acetyl-CoA is converted to malonyl-CoA by ACC or to cholesterol by HMG-CoA synthetase. Studies on animal models clearly show that genetic or pharmacological inhibition of ACLY acts on hepatic steatosis by controlling lipogenesis (21, 23, 34). In this context, inhibition of ACLY by bempedoic acid in hepatic stellate cells impaired their activation and proliferation reducing fibrosis and progression of NAFLD to NASH in mice (21). The action of bempedoic acid on hepatic steatosis as observed in animal models should be confirmed by clinical studies in humans. Indeed, hepatic *de novo* synthesis rates of fatty acids are relatively high in rodents, while in normal humans less than 10% of intrahepatic triglyceride accumulation arises from fatty acid synthesis (35).

### Diabetes mellitus

Evidence from meta-analyses of RCTs demonstrates that statin therapy increases the risk of new-onset diabetes by around 12%. Statins may affect beta-cell function, downregulate glucagon-like peptide 1 receptor and promote insulin resistance in skeletal muscle (36). Regarding the PCSK9 inhibitors, RCTs have clearly shown that alirocumab or evolocumab administration does not lead to new-onset diabetes or aggravate pre-existent type 2 diabetes mellitus (36). Unlike statins and PCSK9 inhibitors, bempedoic acid shows a clear potential positive effect on glucose metabolism and insulin sensitivity *via* the activation of AMPK, which leads to the consequent inhibitory actions on the metabolic pathways regulated downstream. The main effect consists in the inhibition of the hepatic production of glucose through the inhibition of the enzymes G6Pase and PEPCK. Metformin, the first-line drug for type 2 diabetes treatment and prevention, shares a stimulatory effect on the AMPK pathway with bempedoic acid. The primary action of metformin is decreasing gluconeogenesis and hepatic glucose production. Phase III RCT on bempedoic acid included about 20–30% type 2 diabetic patients in whom hemoglobin A1c (HbA1c) decreased by 0.12% as compared to HbA1c ≥ 6.5% (48 mmol/mol) at baseline (37). A meta-analysis showed significantly lower rates of new-onset or worsening of type 2 diabetes mellitus with bempedoic acid (OR 0.68; *p* = 0.02) (38). The cholesterol-lowering response to bempedoic acid was not different in diabetic and in non-diabetic patients (39, 40).

### Inflammation

It is widely accepted that the beneficial effects of statins on cardiovascular events are mainly linked to their cholesterol-lowering properties. A meta-analysis of 14 RCTs with 90,000 subjects demonstrated that approximately 1 mmol/L reduction in LDL-C results in approximately 20% reduction in major vascular events. Nonetheless, statins also have a clear action in reducing systemic inflammation. The effect on hsCRP is proportionally similar to that on LDL-C. It is known that an anti-inflammatory action contributes to the reduction of cardiovascular risk independently of the effect on LDL-C. Four-year therapy with canakinumab, a specific anti-interleukin-1β monoclonal antibody, in patients with previous myocardial infarction reduced the relative risk of relapse by approximately 15% by reducing hsCRP levels by 60% without modifying LDL-C (41). Those patients achieving hsCRP concentrations <2 mg/L showed the greatest benefits on cardiovascular mortality (42). Recent RCTs indicate that to obtain the greatest reduction in cardiovascular events, it is necessary to reach both LDL-C (i.e., <70 or <55 mg/dl) and hrCRP (i.e., <2 mg/L) (43). It is therefore possible to hypothesize that cholesterol-lowering drugs with an effect on hsCRP, such as statins and bempedoic acid, have advantages in reducing cardiovascular events compared to those that do not act on inflammation, such as PCSK9 inhibitors. Bempedoic acid administration is associated with a systemic anti-inflammatory action similar to that of statins. In phase III RCTs, bempedoic acid administration was associated with 20–30% decrease in hsCRP levels, reducing inflammation and possibly the risk of ASCVD (44). The anti-inflammatory action of bempedoic acid is mainly achieved *via* activation of AMPK and inhibition of the MAPK pathway in the immune cells. In a mouse model, activation of the AMPK pathway by bempedoic acid reduced chemokine, cytokine, and adhesion molecule secretion as well as leukocyte accumulation and activation in artery subendothelium and visceral adipose tissues (24). Through these mechanisms, bempedoic acid may prevent atherosclerosis and type 2 diabetes.

### Atherosclerosis

Preclinical models have indicated that bempedoic acid inhibits atherosclerosis and this mechanism may be mainly due to the suppression of inflammation and the regulation of lipid metabolism, especially lowering LDL-C. Samsoondar et al. reported that in *LDLR*^–/^*^–^* mice, subjected to a high-fat diet, bempedoic acid supplementation attenuated aortic inflammation, reducing the expression of pro-inflammatory genes, and decreased lipid levels in the plasma and their accumulation in liver and aortic wall, subsequently preventing the development of atherosclerotic lesions (23). Therefore, another study demonstrated in high-fat diet-fed large animal models of familial hypercholesterolemia, that the treatment with bempedoic acid reduced plasma cholesterol and LDL-C and hampered the development of coronary and aortic atherosclerosis. In *LDLR*^±^ minipigs, bempedoic acid decreased left anterior descending coronary artery lesion area (−40%) and aortic lesion area (−58%), whereas in *LDLR*^–/^*^–^* minipigs left anterior descending coronary artery lesion area (−48%) and aortic lesion area (−47%) (45). Nonetheless, phase III clinical trials concluded to date have not definitively demonstrated the efficacy of bempedoic in reducing cardiovascular and total mortality and cardiovascular events. These trials were not specifically designed with these goals. A large clinical trial designed specifically to demonstrate the effects of bempedoic on these primary outcomes is currently underway.

## Phase III trials testing bempedoic acid for clinical safety and outcomes

Four phase III RCTs on Cholesterol Lowering *via* bempedoic acid, an ACLY-Inhibiting Regimen (CLEAR) have been completed in recent years (Table 2) (46–49). The primary objective was to demonstrate the safety (50) of the treatment while the secondary objective was to demonstrate the reduction effects on LDL-C. CLEAR Harmony (NCT02666664) (48) and CLEAR Wisdom (NCT02991118) (47) enrolled patients with ASCVD and/or HeFH and persistent hypercholesterolemia despite statin therapy at the maximum tolerated dose. CLEAR Tranquility (NCT03001076) (46) and CLEAR Serenity (NCT02988115) (49) enrolled patients with ASCVD and/or HeFH or primary prevention and statin intolerance. A meta-analysis of pooled results of the CLEAR RCTs, including 3,623 patients, was conducted to define safety of bempedoic acid versus placebo (51). Common adverse events occurred at similar rates in the two groups showing a high degree of safety. Bempedoic acid was associated with modest and reversible increases in blood uric acid levels and a 2.5-fold greater incidence of gout. Previous episodes of gout is a risk factor for the development of acute gout during therapy with bempedoic acid. Small increases in blood creatinine levels (<0.05 mg/dl) have been reported during therapy with bempedoic acid, which were stable and reversible upon discontinuation of treatment (38). Small decreases in estimate glomerular filtration rate (eGFR) were also observed during bempedoic acid therapy. Bempedoic acid is a weak inhibitor of the tubular transporter of type 2 organic acids (OAT2) which contributes to uric acid and creatinine secretion by proximal tubular cells (52, 53). The low-grade and reversible increases in plasma uric acid and creatinine associated with bempedoic acid therapy are due to competition for OAT2 in the renal tubules. Thus, small decreases in eGFR are secondary to drug-induced reduction in tubular secretion of creatinine. Bempedoic acid may lead to mild, asymptomatic and reversible increases in alanine aminotransferase and aspartate aminotransferase levels. Bempedoic acid therapy was also associated with a tendency for tendon disorders, which occurred in 0.5% of patients. Tendon injury mainly involved Achilles tendon, rotator cuff and biceps tendon. All tendon injuries occurred in patients taking moderate or high dose statins. No tendon injury occurred in statin intolerant patients who were treated with bempedoic acid. In addition to moderate or high dose statins, other risk factors for tendon injury include fluoroquinolone or corticosteroid therapy, gout, diabetes, rheumatoid arthritis, renal failure, aging, and male sex. Phase III RCTs indicates that compared with placebo, bempedoic acid therapy is not associated with myalgia and muscle weakness even in patients with statin intolerance. No cases of rhabdomyolysis were observed (50).

**TABLE 2:** Phase 3 clinical trials of bempedoic acid.

	CLEAR Harmony (48)	CLEAR Wisdom (47)	CLEAR Serenity (49)	CLEAR Tranquility (46)
Clinical trial number	NCT02666664	NCT02991118	NCT02988115	NCT03001076
Patient population	*N* = 2230	*N* = 779	*N* = 345	*N* = 269
	ASCVD 98%	ASCVD 94%	ASCVD 39%	
	HeFH 4%	HeFH 5%	HeFH 2%	
	HTN 79%	HTN 85%	HTN 68%	HTN 60%
	DM 29%	DM 30%	DM 26%	DM 19%
Background therapy	Maximally tolerated statin therapy	Maximally tolerated statin therapy	Statin intolerance and hypercholesterolemia on low or no statin therapy	Statin intolerance and hypercholesterolemia on low or no statin therapy
Participants (BA/P)	1,488/742	522/257	234/111	181/88
Primary objective	Incidence of treatment related adverse effects	% change in LDL-C after 12 weeks of therapy	% change in LDL-C after 12 weeks of therapy	% change in LDL-C after 12 weeks of therapy
Daily dose (mg)	180	180	180	180
Duration of treatment (weeks)	52	12	24	12
Baseline LDL-C (mean, mg/dl) (BA/P)	104 ± 29/102 ± 30	119 ± 38/122 ± 38	159 ± 40/156 ± 39	130 ± 31/123 ± 27
LDL-C change (%) (BA/P)	−16.5/+1.6	−15.1/+2.4	−23.6/−1.3	−23.5/+5.0
Baseline hsCRP (median, mg/L) (BA/P)	1.5/1.5	1.6/1.9	2.9/2.8	2.2/2.3
hsCRP change (%, BA/P)	−22.4/+2.6	−18.7/−9.4	−25.4/+2.7	−32.5/+2.1
Muscular disorder (%, BA/P)	13.1/10.1	7.5/5.1	12.8/16.2	1.7/2.3
MACE (%, BA/P)	4.6/5.7	5.4/7.8	3.8/0	–
New-onset or worsening of DM (%, BA/P)	3.3/5.4	6.9/7.4	2.1/4.5	1.1/2.3
Uric acid change (mg/dl, BA/P)	+0.7/−0.1	+0.6/+0.1	+0.9/−0.1	+0.6/?
Gout (%, BA/P)	1.2/0.3	2.1/0.8	1.7/0.9	0/0

ASCVD, atherosclerotic cardiovascular disease; DM, diabetes mellitus; HeFH, heterozygous familial hypercholesterolemia; HTN, hypertension; LDL-C, low-density lipoprotein-cholesterol; BA/P, bempedoic acid versus placebo; hsCRP, high-sensitivity C-reactive protein; MACE, Major Adverse Cardiovascular Events.

In order to assess the effect of bempedoic acid on clinical outcomes the phase III studies completed so far have been analyzed with the technique of meta-analysis (38). Six RCTs were identified with a total of 3,956 patients and follow-up from 4 to 52 weeks (28, 46–49, 54). A high heterogeneity, mainly deriving from the different duration of follow-up and from the variability of the basic cardiovascular risk, made the results on clinical patients difficult to interpret. No difference was observed for bempedoic acid compared to usual standard therapy with regard to the composite cardiovascular MACE, mortality from all causes and cardiovascular mortality. Bempedoic acid has shown positive trends in reducing the incidence of non-fatal myocardial infarction. Nonetheless, current RCTs have not demonstrated the ability of bempedoic acid to prevent all-cause and cardiovascular mortality because none of these studies was designed for these goals. The fact that a small number of events were observed in these studies increased the likelihood of a type II statistical error. With this aim, the CLEAR Outcomes study (NCT02993406) is underway (55), which has as its primary objective the evaluation of the effect of administering bempedoic acid for about 3.5 years on cardiovascular outcomes. About 14,000 high-risk cardiovascular patients intolerant to statins will be enrolled. The CLEAR Outcomes study will also provide additional insights on the bempedoic acid safety profile. The results of this study are expected by 2023.

### Pharmacokinetics of bempedoic acid

Cicero et al. recently reviewed the pharmacokinetics of bempedoic acid in the treatment of hypercholesterolemia (56). The recommended daily dose is 180 mg tablet, taken orally with meals or between meals. The elimination of bempedoic acid and bempedoyl-CoA is mainly (70%) carried out by conjugation with glucuronic acid and renal excretion. Liver elimination is responsible for about 30% of bempedoic acid clearance. In mild to moderate hepatic impairment (Child-Pugh class A and B), no dosage adjustment is required (57). Bempedoic acid should not be administered to patients with severe liver disease (Child-Pugh class C). Patients with chronic kidney disease generally well tolerate bempedoic acid therapy, regardless of the degree of renal impairment. Thus, no dose adjustments are currently required for patients with mild or moderate renal impairment (58). Bempedoic acid has not been sufficiently studied in patients with severe renal impairment (eGFR <30 ml/min/1.73 m^2^) or in those with end-stage renal disease on dialysis. Pharmacokinetic drug-drug interactions between bempedoic acid and simvastatin, atorvastatin, pravastatin, rosuvastatin, and ezetimibe were evaluated in clinical studies. Results indicate that the co-administration of bempedoic acid with simvastatin or pravastatin significantly increases the area under the curve (AUC) and C_max_ of the two statins, while there are no significant drug–drug interaction with atorvastatin, rosuvastatin, and ezetimibe, except for slight elevations of the AUCs. Consequently, the risk of developing myalgia or myopathy increases with the combined use of bempedoic acid with simvastatin or pravastatin >20 mg and >40 mg, respectively. No dosage adjustment is necessary in case of combined administration of bempedoic acid with atorvastatin, rosuvastatin or ezetimibe.

### Management of patients receiving bempedoic acid therapy

In light of the efficacy and safety results obtained in phase II and III RCTs, bempedoic acid alone or in combination with ezetimibe has received US FDA and EMA approval for use in adults with HeFH and/or established ASCVD (Table 3) (44, 57). Bempedoic acid administration is aimed at achieving target LDL-C levels in patients with or without statin intolerance. The drug can be administered alone or in combination with other LDL-C lowering drugs, such as ezetimibe, statins, or PCSK9 inhibitors. As predicted by the mechanism of action, administration of bempedoic acid in monotherapy is not significantly associated with myalgia, muscle weakness, or myopathy. Nonetheless, bempedoic acid increases statin plasma concentrations, mainly simvastatin and pravastatin and to a lesser extent atorvastatin and rosuvastatin. Bempedoic acid should not be administered in combination with simvastatin doses higher than 20 mg/day or pravastatin doses higher than 40 mg/day (Table 3). Patients receiving bempedoic acid as add-on therapy to a high-intensity statin (i.e., atorvastatin 40–80 mg/day or rosuvastatin 20–40 mg/day) should be monitored in order to detect any adverse reactions associated with the use of high doses of statins, especially statin related myotoxicity (Table 3). No dosage adjustment is necessary in mild to moderate chronic kidney disease (i.e., stage 1 to 3, eGFR >30 ml/min/1.73 m^2^) or hepatic impairment (i.e., Child-Pugh class A and B) (Table 3). There are no data about its use in pregnancy and lactation. In patients at increased risk for hyperuricemia and gout uric acid should be assessed. Patient at risk of tendon injury (i.e., >60 years of age, taking corticosteroids or fluoroquinolones, having renal failure or arthritis) should be advised to rest at first sign of tendon inflammation or rupture.

**TABLE 3:** Practical management of patients receiving bempedoic acid.

Use	Treatment of established ASCVD and/or HeFH, in addition to diet and/or maximally tolerated statin therapy in adult patients who require additional lowering of LDL-C.
Dose	180 mg/day
Adverse reactions	Hyperuricemia and gout (2.5-fold greater incidence); assess serum uric acid in all patients especially in those with prior history or risk factors for gout. Rupture of tendon (0.5%); discontinue in those taking corticosteroids or fluoroquinolones or with tendon inflammation or with history of tendon rupture.
Dosage adjustment in comorbidities	No dosage adjustment is necessary in mild to moderate chronic kidney disease (i.e., stage 1 to 3, eGFR >30 ml/min/1.73 m^2^) or hepatic impairment (i.e., Child-Pugh class A and B).
Drug-drug interactions in combination therapy	Ezetimibe: 10 mg/day, no drug-drug interactions. Statins: bempedoic acid increases statin plasma concentrations with greater incidence of myalgia and myopathy; avoid taking simvastatin dose >20 mg or pravastatin >40 mg, less interaction with atorvastatin and rosuvastatin. Alirocumab and Evolocumab: no drug-drug interactions.

ASCVD, atherosclerotic cardiovascular disease; HeFH, heterozygous familial hypercholesterolemia; LDL-C, low-density lipoprotein cholesterol; eGFR, estimate glomerular filtration rate.

## Author contributions

GB, AM, and FDG wrote the manuscript. PV, ML, PS, NF, and FM critically reviewed the manuscript. GB and AM prepared figure and tables and edited the manuscript. All authors contributed to the article and approved the submitted version.

## References

[1] Authors/Task Force Members, ESC Committee for Practice Guidelines (CPG), ESC National Cardiac Societies. 2019 ESC/EAS guidelines for the management of dyslipidaemias: lipid modification to reduce cardiovascular risk. *Atherosclerosis.* (2019) 290:140–205. 10.1016/j.atherosclerosis.2019.08.014 31591002

[2] Mach F Baigent C Catapano AL Koskinas KC Casula M Badimon L 2019 ESC/EAS Guidelines for the management of dyslipidaemias: lipid modification to reduce cardiovascular risk. *Eur Heart J.* (2020) 41:111–88. 10.1093/eurheartj/ehz455 31504418

[3] Laslett LJ Alagona P Jr Clark BA III Drozda JP Jr Saldivar F Wilson SR The worldwide environment of cardiovascular disease: prevalence, diagnosis, therapy, and policy issues: a report from the American College of Cardiology. *J Am Coll Cardiol.* (2012) 60(25 Suppl.):S1–49. 10.1016/j.jacc.2012.11.002 23257320

[4] EAS Familial Hypercholesterolaemia Studies Collaboration [FHSC]. Global perspective of familial hypercholesterolaemia: a cross-sectional study from the EAS familial hypercholesterolaemia studies collaboration (FHSC). *Lancet.* (2021) 398:1713–25. 10.1016/S0140-6736(21)01122-3 34506743

[5] Rizos CV Skoumas I Rallidis L Skalidis E Tziomalos K Garoufi A LDL cholesterol target achievement in heterozygous familial hypercholesterolemia patients according to 2019 ESC/EAS lipid guidelines: implications for newer lipid-lowering treatments. *Int J Cardiol.* (2021) 345:119–24. 10.1016/j.ijcard.2021.10.024 34687802

[6] van de Borne P Peeters A Janssens L Leone A Lemmens R Verhaegen A Lipid-lowering therapy and risk-based LDL-C goal attainment in Belgium: DA VINCI observational study. *Acta Cardiol.* (2022) 1–10. 10.1080/00015385.2022.2030568 35442151

[7] Ofori-Asenso R Zoungas S Liew D. Reinitiation of statin therapy after discontinuation: a meta-analysis. *Mayo Clin Proc.* (2018) 93:666–8. 10.1016/j.mayocp.2018.01.011 29728206

[8] Vinci P Panizon E Tosoni LM Cerrato C Pellicori F Mearelli F Statin-associated myopathy: emphasis on mechanisms and targeted therapy. *Int J Mol Sci.* (2021) 22:11687. 10.3390/ijms222111687 34769118 PMC8583847

[9] Casula M Gazzotti M Bonaiti F OImastroni E Arca M Averna M Reported muscle symptoms during statin treatment amongst Italian dyslipidaemic patients in the real-life setting: the PROSISA Study. *J Intern Med.* (2021) 290:116–28. 10.1111/joim.13219 33259671 PMC8359216

[10] Thompson PD Zmuda JM Domalik LJ Zimet RJ Staggers J Guyton JR. Lovastatin increases exercise-induced skeletal muscle injury. *Metabolism.* (1997) 46:1206–10. 10.1016/s0026-0495(97)90218-3 9322808

[11] Parker BA Augeri AL Capizzi JA Ballard KD Troyanos C Baggish AL Effect of statins on creatine kinase levels before and after a marathon run. *Am J Cardiol.* (2012) 109:282–7. 10.1016/j.amjcard.2011.08.045 22036108

[12] Di Girolamo FG Situlin R Fiotti N Tence M De Colle P Mearelli F Higher protein intake is associated with improved muscle strength in elite senior athletes. *Nutrition.* (2017) 42:82–6. 10.1016/j.nut.2017.05.003 28870484

[13] Di Girolamo FG Guadagni M Fiotti N Situlin R Biolo G. Contraction and nutrition interaction promotes anabolism in cachectic muscle. *Curr Opin Clin Nutr Metab Care.* (2019) 22:60–7. 10.1097/MCO.0000000000000527 30461449

[14] Di Girolamo FG Fiotti N Milanovic Z Situlin R Mearelli F Vinci P The aging muscle in experimental bed rest: a systematic review and meta-analysis. *Front Nutr.* (2021) 8:633987. 10.3389/fnut.2021.633987 34422875 PMC8371327

[15] Parham JS Goldberg AC. Major concepts in treatment with bempedoic acid and inclisiran that clinicians need to know. *Curr Atheroscler Rep.* (2022) 24:619–25. 10.1007/s11883-022-01036-4 35666408

[16] Mercep I Strikic D Sliskovic AM Reiner Z. New therapeutic approaches in treatment of dyslipidaemia-a narrative review. *Pharmaceuticals.* (2022) 15:839. 10.3390/ph15070839 35890138 PMC9324773

[17] Pinkosky SL Filippov S Srivastava RA Hanselman JC Bradshaw CD Hurley TR AMP-activated protein kinase and ATP-citrate lyase are two distinct molecular targets for ETC-1002, a novel small molecule regulator of lipid and carbohydrate metabolism. *J Lipid Res.* (2013) 54:134–51. 10.1194/jlr.M030528 23118444 PMC3520520

[18] Pinkosky SL Newton RS Day EA Ford RJ Lhotak S Austin RC Liver-specific ATP-citrate lyase inhibition by bempedoic acid decreases LDL-C and attenuates atherosclerosis. *Nat Commun.* (2016) 7:13457. 10.1038/ncomms13457 27892461 PMC5133702

[19] Burke AC Telford DE Huff MW. Bempedoic acid: effects on lipoprotein metabolism and atherosclerosis. *Curr Opin Lipidol.* (2019) 30:1–9. 10.1097/MOL.0000000000000565 30586346

[20] Ference BA Ray KK Catapano AL Ference TB Burgess S Neff DR Mendelian randomization study of ACLY and cardiovascular disease. *N Engl J Med.* (2019) 380:1033–42. 10.1056/NEJMoa1806747 30865797 PMC7612927

[21] Morrow MR Batchuluun B Wu J Ahmadi E Leroux JM Mohammadi-Shemirani P Inhibition of ATP-citrate lyase improves NASH, liver fibrosis, and dyslipidemia. *Cell Metab.* (2022) 34:919–36.e8. 10.1016/j.cmet.2022.05.004 35675800

[22] Bouchard-Mercier A Rudkowska I Lemieux S Couture P Vohl MC. Polymorphisms, de novo lipogenesis, and plasma triglyceride response following fish oil supplementation. *J Lipid Res.* (2013) 54:2866–73. 10.1194/jlr.M041590 23886516 PMC3770099

[23] Samsoondar JP Burke AC Sutherland BG Telford DE Sawyez CG Edwards JY Prevention of diet-induced metabolic dysregulation, inflammation, and atherosclerosis in ldlr(–/–) mice by treatment with the ATP-citrate lyase inhibitor bempedoic acid. *Arterioscler Thromb Vasc Biol.* (2017) 37:647–56. 10.1161/ATVBAHA.116.308963 28153881

[24] Filippov S Pinkosky SL Lister RJ Pawloski C Hanselman JC Cramer CT ETC-1002 Regulates immune response, leukocyte homing, and adipose tissue inflammation via LKB1-dependent activation of macrophage AMPK. *J Lipid Res.* (2013) 54:2095–108. 10.1194/jlr.M035212 23709692 PMC3708360

[25] Verberk SGS Kuiper KL Lauterbach MA Latz E Van den Bossche J. The multifaceted therapeutic value of targeting ATP-citrate lyase in atherosclerosis. *Trends Mol Med.* (2021) 27:1095–105. 10.1016/j.molmed.2021.09.004 34635427

[26] Thompson PD Rubino J Janik MJ MacDougall DE McBride SJ Margulies JR Use of ETC-1002 to treat hypercholesterolemia in patients with statin intolerance. *J Clin Lipidol.* (2015) 9:295–304. 10.1016/j.jacl.2015.03.003 26073387

[27] Thompson PD MacDougall DE Newton RS Margulies JR Hanselman JC Orloff DG Treatment with ETC-1002 alone and in combination with ezetimibe lowers LDL cholesterol in hypercholesterolemic patients with or without statin intolerance. *J Clin Lipidol.* (2016) 10:556–67. 10.1016/j.jacl.2015.12.025 27206943

[28] Gutierrez MJ Rosenberg NL Macdougall DE Hanselman JC Margulies JR Strange P Efficacy and safety of ETC-1002, a novel investigational low-density lipoprotein-cholesterol-lowering therapy for the treatment of patients with hypercholesterolemia and type 2 diabetes mellitus. *Arterioscler Thromb Vasc Biol.* (2014) 34:676–83. 10.1161/ATVBAHA.113.302677 24385236

[29] Lalwani ND Hanselman JC MacDougall DE Sterling LR Cramer CT. Complementary low-density lipoprotein-cholesterol lowering and pharmacokinetics of adding bempedoic acid (ETC-1002) to high-dose atorvastatin background therapy in hypercholesterolemic patients: a randomized placebo-controlled trial. *J Clin Lipidol.* (2019) 13:568–79. 10.1016/j.jacl.2019.05.003 31202641

[30] Rubino J MacDougall DE Sterling LR Hanselman JC Nicholls SJ. Combination of bempedoic acid, ezetimibe, and atorvastatin in patients with hypercholesterolemia: a randomized clinical trial. *Atherosclerosis.* (2021) 320:122–8. 10.1016/j.atherosclerosis.2020.12.023 33514449

[31] Rubino J MacDougall DE Sterling LR Kelly SE McKenney JM Lalwani ND. Lipid lowering with bempedoic acid added to a proprotein convertase subtilisin/kexin type 9 inhibitor therapy: a randomized, controlled trial. *J Clin Lipidol.* (2021) 15:593–601. 10.1016/j.jacl.2021.05.002 34172394

[32] Cicero AFG Pontremoli R Fogacci F Viazzi F Borghi C. Effect of bempedoic acid on serum uric acid and related outcomes: a systematic review and meta-analysis of the available phase 2 and phase 3 clinical studies. *Drug Saf.* (2020) 43:727–36. 10.1007/s40264-020-00931-6 32358698

[33] Sanders FWB Acharjee A Walker C Marney L Roberts LD Imamura F Hepatic steatosis risk is partly driven by increased de novo lipogenesis following carbohydrate consumption. *Genome Biol.* (2018) 19:79. 10.1186/s13059-018-1439-8 29925420 PMC6009819

[34] Sanjay KV Vishwakarma S Zope BR Mane VS Mohire S Dhakshinamoorthy S. ATP citrate lyase inhibitor Bempedoic Acid alleviate long term HFD induced NASH through improvement in glycemic control, reduction of hepatic triglycerides & total cholesterol, modulation of inflammatory & fibrotic genes and improvement in NAS score. *Curr Res Pharmacol Drug Discov.* (2021) 2:100051. 10.1016/j.crphar.2021.100051 34909677 PMC8663992

[35] Postic C Girard J. Contribution of de novo fatty acid synthesis to hepatic steatosis and insulin resistance: lessons from genetically engineered mice. *J Clin Invest.* (2008) 118:829–38. 10.1172/JCI34275 18317565 PMC2254980

[36] Xiao X Luo Y Peng D. Updated understanding of the crosstalk between glucose/insulin and cholesterol metabolism. *Front Cardiovasc Med.* (2022) 9:879355. 10.3389/fcvm.2022.879355 35571202 PMC9098828

[37] Leiter LA Banach M Catapano AL Duell PB Gotto AM Jr Laufs U Bempedoic acid in patients with type 2 diabetes mellitus, prediabetes, and normoglycaemia: a post hoc analysis of efficacy and glycaemic control using pooled data from phase 3 clinical trials. *Diabetes Obes Metab.* (2022) 24:868–80. 10.1111/dom.14645 34981622 PMC9306638

[38] Lin Y Parco C Karathanos A Krieger T Schulze V Chernyak N Clinical efficacy and safety outcomes of bempedoic acid for LDL-C lowering therapy in patients at high cardiovascular risk: a systematic review and meta-analysis. *BMJ Open.* (2022) 12:e048893. 10.1136/bmjopen-2021-048893 35210334 PMC8883220

[39] Masson W Barbagelata L Lobo M Nogueira JP. Bempedoic acid in patients with type 2 diabetes. *Rev Clin Esp.* (2022) 222:251–3. 10.1016/j.rceng.2021.11.003 35241414

[40] Wang X Zhang Y Tan H Wang P Zha X Chong W Efficacy and safety of bempedoic acid for prevention of cardiovascular events and diabetes: a systematic review and meta-analysis. *Cardiovasc Diabetol.* (2020) 19:128. 10.1186/s12933-020-01101-9 32787939 PMC7425167

[41] Ridker PM Everett BM Thuren T MacFadyen JG Chang WH Ballantyne C Antiinflammatory therapy with canakinumab for atherosclerotic disease. *N Engl J Med.* (2017) 377:1119–31. 10.1056/NEJMoa1707914 28845751

[42] Ridker PM MacFadyen JG Everett BM Libby P Thuren T Glynn RJ Relationship of C-reactive protein reduction to cardiovascular event reduction following treatment with canakinumab: a secondary analysis from the CANTOS randomised controlled trial. *Lancet.* (2018) 391:319–28. 10.1016/S0140-6736(17)32814-3 29146124

[43] Ruscica M Ferri N Macchi C Corsini A Sirtori CR. Lipid lowering drugs and inflammatory changes: an impact on cardiovascular outcomes? *Ann Med.* (2018) 50:461–84. 10.1080/07853890.2018.1498118 29976096

[44] Ballantyne CM Bays H Catapano AL Goldberg A Ray KK Saseen JJ. Role of bempedoic acid in clinical practice. *Cardiovasc Drugs Ther.* (2021) 35:853–64. 10.1007/s10557-021-07147-5 33818688 PMC8266788

[45] Burke AC Telford DE Sutherland BG Edwards JY Sawyez CG Barrett PHR Bempedoic acid lowers low-density lipoprotein cholesterol and attenuates atherosclerosis in low-density lipoprotein receptor-deficient (LDLR(+/–) and LDLR(–/–)) yucatan miniature pigs. *Arterioscler Thromb Vasc Biol.* (2018) 38:1178–90. 10.1161/ATVBAHA.117.310676 29449335

[46] Ballantyne CM Banach M Mancini GBJ Lepor NE Hanselman JC Zhao X Efficacy and safety of bempedoic acid added to ezetimibe in statin-intolerant patients with hypercholesterolemia: a randomized, placebo-controlled study. *Atherosclerosis.* (2018) 277:195–203. 10.1016/j.atherosclerosis.2018.06.002 29910030

[47] Goldberg AC Leiter LA Stroes ESG Baum SJ Hanselman JC Bloedon LT Effect of bempedoic acid vs placebo added to maximally tolerated statins on low-density lipoprotein cholesterol in patients at high risk for cardiovascular disease: the CLEAR wisdom randomized clinical trial. *JAMA.* (2019) 322:1780–8. 10.1001/jama.2019.16585 31714986 PMC6865290

[48] Ray KK Bays HE Catapano AL Lalwani ND Bloedon LT Sterling LR Safety and efficacy of bempedoic acid to reduce LDL cholesterol. *N Engl J Med.* (2019) 380:1022–32. 10.1056/NEJMoa1803917 30865796

[49] Laufs U Banach M Mancini GBJ Gaudet D Bloedon LT Sterling LR Efficacy and safety of bempedoic acid in patients with hypercholesterolemia and statin intolerance. *J Am Heart Assoc.* (2019) 8:e011662. 10.1161/JAHA.118.011662 30922146 PMC6509724

[50] Bays HE Banach M Catapano AL Duell PB Gotto AM Jr Laufs U Bempedoic acid safety analysis: pooled data from four phase 3 clinical trials. *J Clin Lipidol.* (2020) 14:649–59.e6. 10.1016/j.jacl.2020.08.009 32980290

[51] Banach M Duell PB Gotto AM Jr Laufs U Leiter LA Mancini GBJ Association of bempedoic acid administration with atherogenic lipid levels in phase 3 randomized clinical trials of patients with hypercholesterolemia. *JAMA Cardiol.* (2020) 5:1124–35. 10.1001/jamacardio.2020.2314 32609313 PMC7330832

[52] Sato M Mamada H Anzai N Shirasaka Y Nakanishi T Tamai I. Renal secretion of uric acid by organic anion transporter 2 (OAT2/SLC22A7) in human. *Biol Pharm Bull.* (2010) 33:498–503. 10.1248/bpb.33.498 20190416

[53] Nakada T Kudo T Kume T Kusuhara H Ito K. Estimation of changes in serum creatinine and creatinine clearance caused by renal transporter inhibition in healthy subjects. *Drug Metab Pharmacokinet.* (2019) 34:233–8. 10.1016/j.dmpk.2019.02.006 31176593

[54] Ballantyne CM Laufs U Ray KK Leiter LA Bays HE Goldberg AC Bempedoic acid plus ezetimibe fixed-dose combination in patients with hypercholesterolemia and high CVD risk treated with maximally tolerated statin therapy. *Eur J Prev Cardiol.* (2020) 27:593–603. 10.1177/2047487319864671 31357887 PMC7153222

[55] Nicholls S Lincoff AM Bays HE Cho L Grobbee DE Kastelein JJ Rationale and design of the CLEAR-outcomes trial: evaluating the effect of bempedoic acid on cardiovascular events in patients with statin intolerance. *Am Heart J.* (2021) 235:104–12. 10.1016/j.ahj.2020.10.060 33470195

[56] Cicero AFG Fogacci F Cincione I. Evaluating pharmacokinetics of bempedoic acid in the treatment of hypercholesterolemia. *Expert Opin Drug Metab Toxicol.* (2021) 17:1031–8. 10.1080/17425255.2021.1951222 34197267

[57] Averna M Bilato C Sesti G Italian Clinical Network on Bempedoic Acid. Clinical evaluation of bempedoic acid for the treatment of hyperlipidaemia. . *Nutr Metab Cardiovasc Dis.* (2022) 32:17–20. 10.1016/j.numecd.2021.09.023 34802854

[58] Amore BM Sasiela WJ Ries DK Tresh P Emery MG. Pharmacokinetics of bempedoic acid in patients with renal impairment. *Clin Transl Sci.* (2022) 15:789–98. 10.1111/cts.13202 34800002 PMC8932715

